# Prevention and Management of Compassion Fatigue in Clinical Nurses: An Evidence Summary

**DOI:** 10.1155/jonm/5171257

**Published:** 2026-11-02

**Authors:** Yuying Lin, Jinyan Wu, Yuan Chen, Shunfang Zhu, Qiuhua Deng

**Affiliations:** ^1^ Department of Respiratory and Critical Care Medicine Nanfang Hospital Southern Medical University Guangzhou 510515 China fimmu.com; ^2^ Department of Nursing Nanfang Hospital Southern Medical University Guangzhou 510515 China fimmu.com; ^3^ Department of Cardiology Nanfang Hospital Southern Medical University Guangzhou 510515 China fimmu.com

**Keywords:** compassion fatigue, evidence-based nursing, hospital, nursing staff, occupational health, occupational health services

## Abstract

**Aim:**

To synthesize and translate available evidence on preventing and managing compassion fatigue (CF) in clinical nurses.

**Background:**

CF threatens nurse well‐being, retention, care quality, and workforce sustainability, yet prevention and management guidance remains fragmented across evidence types.

**Evaluation:**

Using a hierarchical evidence model, databases, evidence resources, and organizational sources were searched from inception to 30 June 2025. Eligible guidelines, consensus statements, systematic reviews or meta‐analyses, and an educational toolkit were independently screened, appraised, extracted, and integrated using predefined weighting criteria.

**Key Issues:**

Of 1481 records, 15 documents were included: three clinical practice guidelines, one educational toolkit, one expert consensus statement, and 10 systematic reviews or meta‐analyses. Sixteen evidence statements were synthesized across six domains. Methodological confidence in the included reviews was moderate for seven and low for three.

**Conclusion):**

CF management requires coordinated individual, team, and organizational strategies, with recommendations adapted to local clinical and cultural contexts.

**Implications for Nursing Management:**

Nurse managers should embed routine risk assessment, targeted education and support, team‐based interventions, workload management, and accessible psychological support within nursing governance.

## 1. Introduction

Compassion fatigue (CF) is a work‐related phenomenon associated with repeated empathic engagement with patients’ suffering and trauma. It is often discussed alongside burnout and secondary traumatic stress (STS), but the constructs are not interchangeable: burnout mainly reflects chronic occupational strain, whereas STS describes trauma‐related symptoms following indirect exposure to others’ traumatic experiences [1, 2]. Conceptual ambiguity can therefore lead to inconsistent measurement and intervention targets. Clinical nurses repeatedly encounter suffering, death, trauma, high workload, and emotionally demanding care. Reported CF levels vary across specialties and studies because populations, settings, instruments, and cutoffs differ [3–5]. This variability supports standardized assessment with context‐sensitive interpretation rather than reliance on a single prevalence estimate. CF and related occupational distress are associated with poorer psychological well‐being, sleep disturbance, reduced professional quality of life, turnover intention, and workforce instability [6–8]. Nurses in emergency, oncology, psychiatric, intensive care, and trauma‐related settings may face particularly frequent distressing exposure [9–11]. Prevention therefore requires individual, team, and organizational responses. Evidence on CF prevention and management is distributed across guidelines, consensus statements, systematic reviews, meta‐analyses, and organizational guidance. Existing syntheses commonly focus on one intervention, outcome, or setting, whereas nurse managers need integrated recommendations that acknowledge methodological quality and directness [12–14]. This study therefore aimed to retrieve, critically appraise, and synthesize evidence on preventing and managing CF in clinical nurses and to formulate practice‐oriented recommendations for nursing management. The evidence summary was registered with the Fudan University Center for Evidence‐Based Nursing (ES20246343).

## 2. Methods

### 2.1. Problem Identification and Operational Framework

Because evidence summaries have no single universally accepted methodological or reporting format, we used the Fudan University Center for Evidence‐Based Nursing procedures as an operational framework for question formulation, hierarchical retrieval, screening, appraisal, extraction, synthesis, and recommendation development. The framework structured the review; it was not treated as a universal reporting standard.

The review integrated heterogeneous sources into practice‐oriented recommendations rather than producing a pooled effect estimate. Eligible sources included clinical practice guidelines, expert consensus, systematic reviews or meta‐analyses, evidence summaries, educational toolkits, and eligible intervention studies. The evidence question was structured using the Population, Intervention, Professionals applying the evidence, Outcomes, Setting, and Type of evidence (PIPOST) framework as follows:

P (Population): registered or clinical nurses providing direct patient care. I (Intervention): prevention, early identification, assessment, education, psychological support, team interventions, and organizational strategies. P (Professionals): nurse managers, occupational‐health staff, mental‐health professionals, hospital administrators, and implementation teams. O (Outcomes): CF severity, STS, burnout, compassion satisfaction, resilience, job satisfaction, turnover intention, sleep quality, perceived stress, and nursing‐care quality. S (Setting): hospitals and other healthcare institutions, including emergency, oncology, intensive care, psychiatric, and trauma‐related settings. T (Type of evidence): guidelines, expert consensus, systematic reviews or meta‐analyses, evidence summaries, educational toolkits, and eligible intervention studies. Point‐of‐care clinical decision documents were not retained in the final synthesis.

### 2.2. Literature Search

Following the 6S evidence model, higher level evidence (guidelines, evidence summaries, consensus documents, systematic reviews, and meta‐analyses) was searched first. Lower level intervention studies were considered only when higher level evidence was unavailable, insufficient, or required contextual supplementation.

We searched, from inception to 30 June 2025, China National Knowledge Infrastructure (CNKI), VIP Chinese Scientific and Technological Journal Database, Chinese Biomedical Literature Database (CBM), Wanfang Data, PubMed, Embase, the Cochrane Library, Web of Science Core Collection, ClinicalKey, Ovid resources, the Joanna Briggs Institute (JBI) Evidence‐Based Healthcare Database, and American Psychological Association (APA) PsycINFO. Targeted organizational and gray‐literature searching covered the Registered Nurses’ Association of Ontario (RNAO), Secondary Traumatic Stress Consortium (STSC), APA, Substance Abuse and Mental Health Services Administration (SAMHSA), and Walden University ScholarWorks. Searches covered database inception to June 30, 2025. English/Chinese eligibility was applied during study selection, and reference lists of potentially eligible and included sources were checked manually.

Searches combined controlled vocabulary, where available, with free‐text terms for (1) CF and related constructs (STS, secondary trauma, vicarious trauma, and emotional exhaustion), (2) nurses/clinical nursing, and (3) eligible evidence types. Platform‐specific field tags, truncation, and Boolean operators were used. Supporting File 1 provides the complete database‐specific strings retained in the search record, search fields, coverage end date, retrieval counts, eligibility limits, organizational/gray‐literature sources, and reference‐list checking procedure. For organizational websites or repositories whose retained search record did not preserve a stable database‐style query syntax, the source, topic focus, and yield are reported without retrospectively reconstructing an unrecorded Boolean string.

### 2.3. Study Inclusion and Exclusion Criteria

#### 2.3.1. Inclusion Criteria

Inclusion criteria were as follows: (i) registered nurses directly involved in clinical care; (ii) prevention, early identification, assessment, or management of CF or closely related occupational‐distress constructs; (iii) at least one predefined primary outcome (CF, STS, burnout, or compassion satisfaction) or secondary outcome (resilience, job satisfaction, turnover intention, sleep quality, perceived stress, or nursing‐care quality); (iv) eligible evidence types comprising guidelines, consensus statements, systematic reviews/meta‐analyses, evidence summaries, best‐practice recommendations, educational toolkits, trials, quasi‐experimental studies, cohort studies, or directly relevant large‐sample observational studies; and (v) full‐text English‐ or Chinese‐language publication. Gray literature additionally had to originate from an authoritative source, be clinically relevant, and provide transparent development or source information.

#### 2.3.2. Exclusion Criteria

Exclusion criteria were as follows: (i) language other than English or Chinese or unavailable full text; (ii) no relevant prevention, identification, assessment, or management content; (iii) prevalence, associated‐factor, or conceptual reports without extractable management strategies; (iv) non‐nurse populations without separately extractable nurse data; (v) ineligible publication types, including narrative reviews, commentaries, case reports, protocols, dissertations, conference abstracts, duplicates, and translated guidelines without original recommendations; or (vi) failure to meet the predefined methodological requirements for synthesis.

### 2.4. Literature Screening

All retrieved records were imported into EndNote, and duplicates were removed. Two reviewers with nursing backgrounds and training in evidence‐based nursing independently screened titles and abstracts and then assessed potentially eligible full texts against the predefined criteria. Methodological appraisal was performed after eligibility assessment. Disagreements at any stage were resolved through discussion; when consensus could not be reached, a senior nursing researcher experienced in evidence‐based practice and nursing management adjudicated the decision.

### 2.5. Quality Appraisal

Clinical practice guidelines were appraised using the Appraisal of Guidelines for Research and Evaluation II (AGREE II). Standardized domain scores were calculated as (obtained score−minimum possible score)/(maximum possible score−minimum possible score) × 100%. Because AGREE II does not prescribe universal recommendation cutoffs, the review used predefined appraisal categories only for transparency: high methodological quality when all six domains were ≥ 60%; moderate quality when at least three domains were 30%–60%; and low quality when at least three domains were < 30%. Systematic reviews/meta‐analyses were appraised with the A Measurement Tool to Assess Systematic Reviews 2 (AMSTAR 2), and overall confidence (high, moderate, low, or critically low) was based on critical and noncritical weaknesses rather than item totals.

Expert consensus was appraised with the six‐item JBI Critical Appraisal Checklist for Text and Opinion. Eligible intervention studies, if required, were appraised with the relevant JBI design‐specific checklist.

No point‐of‐care clinical decision document was retained. The only non‐peer‐reviewed source was an educational toolkit; because no validated appraisal instrument was available for this source type, we assessed source authority, development transparency, clinical relevance, recommendation clarity, and consistency with higher level evidence. It was used only as supplementary evidence.

### 2.6. Evidence Extraction and Synthesis

Two evidence‐trained nurse reviewers independently extracted source, year, evidence type, population, intervention/management strategy, outcomes, findings, evidence level, and recommendation strength using a standardized form; disagreements were resolved with a senior evidence‐based nursing researcher.

Synthesis followed predefined rules: consistent findings were represented by the clearest practice‐oriented wording; complementary findings were integrated; and conflicts were resolved by prioritizing methodological quality, directness, applicability, consistency, and recency.

Evidence was weighted in four tiers. Tier 1 comprised high‐quality guidelines and systematic reviews/meta‐analyses with high or moderate AMSTAR 2 confidence. Tier 2 comprised moderate‐quality guidelines, low‐confidence reviews, and directly relevant well‐designed intervention studies. Tier 3 comprised authoritative consensus statements and evidence summaries. Tier 4 comprised educational toolkits and other eligible gray literature. Lower tier evidence was supplementary and was downgraded when appropriate.

Evidence levels and recommendation strength were assigned using the 2014 JBI systems, considering feasibility, appropriateness, meaningfulness, and effectiveness. Grade A denoted a strong recommendation and Grade B a weaker recommendation. JBI grades were used to express practice recommendation strength and were not treated as equivalent to a formal certainty‐of‐evidence rating.

### 2.7. Ethical Considerations

This evidence summary used only published literature and publicly available evidence sources. It did not involve human participants, patient recruitment, medical records, or identifiable personal data; therefore, ethics approval and informed consent were not required.

## 3. Results

### 3.1. Search Results

Bibliographic database searches yielded 1476 records, and targeted organizational/gray‐literature searches identified five additional records, giving 1481 records before deduplication. After 117 duplicates were removed, 1364 records underwent title/abstract screening, and 956 were excluded. The remaining 408 reports plus five identified through reference checking yielded 413 full texts. Of these, 398 were excluded for ineligible content (*n* = 333), ineligible publication/research type (*n* = 55), or unavailable full text (*n* = 10). Fifteen documents were included: three clinical practice guidelines, one educational toolkit, one expert consensus statement, and 10 systematic reviews/meta‐analyses. The process is shown in Figure 1. Characteristics of the included documents are summarized in Table 1.

**FIGURE 1:**
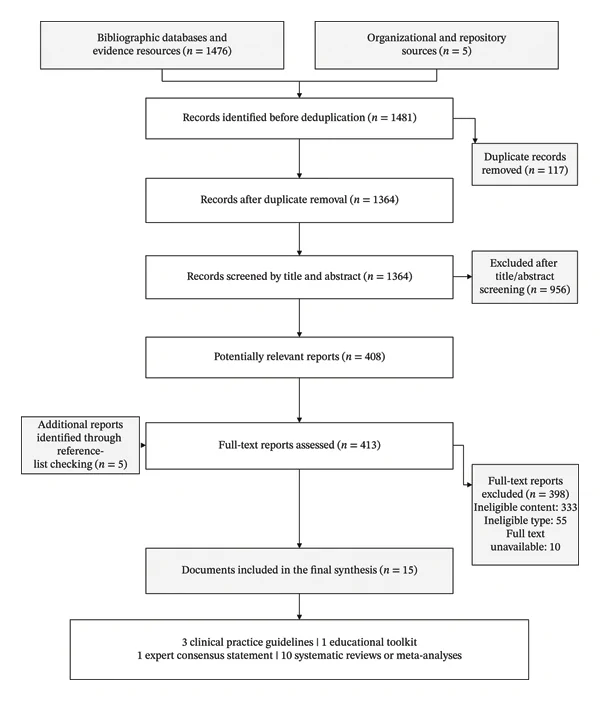
Flow diagram of literature identification, screening, full‐text assessment, and inclusion.

**TABLE 1:** Characteristics of included documents (*n* = 15).

Author/institution	Source	Year	Evidence type	Title/topic
Registered Nurses’ Association of Ontario [15]	RNAO	2011	Best practice guideline	Preventing and Mitigating Nurse Fatigue in Health Care
Secondary Traumatic Stress Consortium [16]	STSC	2021	Best practice guideline	Best Practices for Addressing Secondary Traumatic Stress: Principles for Individuals & Organizations
Bride et al. [17]	APA	2024	Expert consensus statement	Principles for Secondary Traumatic Stress‐Responsive Practice: An Expert Consensus Approach
Substance Abuse and Mental Health Services Administration [18]	SAMHSA	2020	Practical guideline	Tips for Healthcare Professionals: Coping with Stress and Compassion Fatigue
Enakhena [19]	Walden University ScholarWorks	2022	Educational toolkit	Educational Tool Kit for Mental Health Nurses to Combat Compassion Fatigue
Şimşek et al. [20]	PubMed	2025	Systematic review	Investigation of the Effect of Secondary Traumatic Stress on Psychological Resilience in Emergency Nurses: A Systematic Review
Zhang et al. [14]	PubMed	2025	Systematic review and network meta‐analysis	Interventions for Compassion Fatigue, Burnout, and Secondary Traumatic Stress in Nurses: A Systematic Review and Network Meta‐Analysis
Hsu et al. [12]	PubMed	2024	Umbrella review	Effectiveness of Individual‐Based Strategies to Reduce Nurse Burnout: An Umbrella Review
Yu et al. [21]	CNKI	2023	Meta‐analysis	Mindfulness‐Based Therapy for Improving Compassion Fatigue in Nurses: A Meta‐Analysis
Lee & Cha [22]	PubMed	2023	Systematic review and meta‐analysis	Interventions to reduce burnout among clinical nurses: systematic review and meta‐analysis
Wang et al. [23]	Cochrane	2023	Systematic review and meta‐analysis	Effects of Mindfulness‐Based Interventions on Stress and Burnout in Nurses: A Systematic Review and Meta‐Analysis
Chen et al. [24]	Cochrane	2022	Meta‐analysis	Effects of Psychological Intervention on Empathy Fatigue in Nurses: A Meta‐Analysis
Jung et al. [25]	PubMed	2021	Systematic review	The Effectiveness and Safety of Mind‐Body Modalities for Mental Health of Nurses in Hospital Settings: A Systematic Review
Ortega‐Campos et al. [10]	PubMed	2020	Systematic review and meta‐analysis	Compassion Fatigue, Compassion Satisfaction, and Burnout in Oncology Nurses: A Systematic Review and Meta‐Analysis
Patole et al. [13]	PubMed	2024	Systematic review	Interventions for Compassion Fatigue in Healthcare Providers: A Systematic Review of Randomised Controlled Trials

Abbreviations: APA, American Psychological Association; CNKI, China National Knowledge Infrastructure; RNAO, Registered Nurses’ Association of Ontario; SAMHSA, Substance Abuse and Mental Health Services Administration; STSC, Secondary Traumatic Stress Consortium.

### 3.2. Quality Appraisal Results

#### 3.2.1. Quality of Included Guidelines

Three clinical practice guidelines were included. Using the predefined AGREE II appraisal categories, one guideline [15] was classified as high methodological quality and two [16, 18] as moderate methodological quality. The main variation across guidelines concerned rigor of development, stakeholder involvement, applicability, and editorial independence. Detailed domain scores are shown in Table 2. These categories were used only to support transparent appraisal within this evidence summary and do not represent official AGREE II recommendation grades.

**TABLE 2:** AGREE II domain scores and predefined appraisal categories for included guidelines (*n* = 3).

Guideline	Scope and purpose (%)	Stakeholder involvement (%)	Rigour (%)	Clarity (%)	Applicability (%)	Editorial independence (%)	Predefined appraisal category
[15]	85	75	70	80	78	82	High methodological quality
[16]	80	65	55	75	62	45	Moderate methodological quality
[18]	78	50	40	72	68	42	Moderate methodological quality

*Note:* AGREE II = Appraisal of Guidelines for Research and Evaluation II. The appraisal categories were predefined for this evidence summary and are not official AGREE II grading criteria.

Abbreviations: RNAO, Registered Nurses’ Association of Ontario; SAMHSA, Substance Abuse and Mental Health Services Administration; STSC, Secondary Traumatic Stress Consortium.

#### 3.2.2. Quality of Included Expert Consensus

One expert consensus statement [17] was included and appraised using the JBI Critical Appraisal Checklist for Text and Opinion. All six checklist items were rated Yes, including clear identification and standing of the source, explicit focus on the relevant population, a transparent analytical process, reference to the existing literature, and logical treatment of areas of disagreement. The appraisal results are shown in Table 3.

**TABLE 3:** JBI critical appraisal of the included expert consensus statement (*n* = 1).

Items	International expert consensus group on secondary traumatic stress‐responsive practice for behavioral health professionals and organizations [17]
1. Is the source of the opinion clearly identified?	Yes
2. Does the source of opinion have standing in the field of expertise?	Yes
3. Are the interests of the relevant population the central focus of the opinion?	Yes
4. Is the stated position the result of an analytical process, and is there logic in the opinion expressed?	Yes
5. Is there reference to the extant literature?	Yes
6. Is any incongruence with the literature/sources logically defended?	Yes

Abbreviation: JBI, Joanna Briggs Institute.

#### 3.2.3. Quality of Included Systematic Reviews

Ten systematic reviews or meta‐analyses were included and appraised with AMSTAR 2. Seven received a moderate overall confidence rating, and three received a low rating; none was rated high or critically low. Common methodological concerns included incomplete reporting of protocol registration, limited reporting of excluded studies, insufficient description of search resources, uncertainty in duplicate study selection or data extraction, and incomplete assessment of publication bias or certainty. Lower confidence reviews were therefore weighted more cautiously during evidence synthesis and recommendation grading. Table 4 summarizes the overall confidence ratings and key concerns.

**TABLE 4:** AMSTAR 2 overall confidence ratings and key methodological concerns of included systematic reviews and meta‐analyses (*n* = 10).

Included review	Type of review	AMSTAR 2 overall confidence rating	Key methodological concerns
[20]	Systematic review	Moderate	Some limitations in reporting duplicate processes and excluded studies
[14]	Systematic review and network meta‐analysis	Moderate	Limited details on excluded studies and certainty assessment
[12]	Umbrella review	Low	Incomplete reporting of search resources and methodological overlap across included reviews
[21]	Meta‐analysis	Moderate	Limited information on protocol registration and excluded studies
[22]	Systematic review and meta‐analysis	Moderate	Some uncertainty regarding data extraction error control
[23]	Systematic review and meta‐analysis	Moderate	Limited reporting of excluded studies and publication bias handling
[24]	Meta‐analysis	Low	Search resources and protocol information insufficiently reported
[25]	Systematic review	Moderate	Publication bias assessment not applicable or incompletely reported
[10]	Systematic review and meta‐analysis	Low	Search strategy and duplicate appraisal procedures incompletely reported
[13]	Systematic review	Moderate	Some uncertainty in publication bias assessment and practice recommendation support

*Note:* AMSTAR 2 = A Measurement Tool to Assess Systematic Reviews 2. Overall confidence ratings were assigned according to the pattern of critical and noncritical methodological weaknesses. Item‐level judgments informed, but did not replace, the overall confidence rating.

#### 3.2.4. Appraisal of the Educational Toolkit

The educational toolkit was retained as a supplementary source after assessment against the predefined criteria of source authority, transparency, relevance to clinical nursing practice, clarity of recommendations, and consistency with higher level evidence. Because no validated critical appraisal instrument was available for this source type, no formal overall quality grade was assigned; the toolkit was treated as Tier 4 evidence and did not independently determine recommendation strength.

### 3.3. Evidence Summary and Synthesis

Extraction produced 30 candidate statements. After two reviewers independently compared overlapping or complementary content and resolved discrepancies, 16 statements were retained across six domains: cognitive orientation, risk assessment and early identification, proactive prevention, multidimensional interventions, outcome evaluation, and organizational support/implementation. JBI evidence levels and grades were assigned alongside methodological quality, directness, consistency, applicability, and feasibility; lower confidence reviews, consensus, toolkits, and gray literature were interpreted cautiously. Table 5 presents the final synthesis.

**TABLE 5:** Synthesized evidence statements for the prevention and management of compassion fatigue in clinical nurses.

Domain	Synthesized evidence statement	Main supporting sources	Evidence basis/weighting tier	Evidence level	Recommendation grade
Cognitive orientation of management	Compassion fatigue prevention and management should be incorporated into nursing governance and occupational health strategies. Nurse managers should recognize compassion fatigue as an occupational and organizational issue rather than solely an individual coping problem.	[10, 14, 15, 20]	Mainly Tier 1 and Tier 2 evidence: systematic reviews/meta‐analyses and guideline evidence; organizational interpretation applied cautiously	Level 1b	B
	Education should improve nurses’ recognition of compassion fatigue, secondary traumatic stress, burnout, emotional exhaustion, and appropriate help‐seeking. Educational content should also address stigma and psychological resilience.	[14, 18, 20, 21]	Mainly Tier 1 and Tier 3 evidence: systematic reviews supplemented by authoritative guidance; downgraded because implementation details vary	Level 1b	B

Risk assessment and early identification	Risk assessment should prioritize nurses with repeated exposure to trauma, severe illness, death, or high emotional demands, including staff in emergency, intensive care, oncology, psychiatric, trauma‐related, and public‐health‐emergency settings.	[10, 12, 13, 15]	Mainly Tier 1 and Tier 2 evidence: systematic review and guideline evidence; downgraded because risk profiles vary across settings	Level 1b	B
	Standardized, context‐appropriate tools should be selected according to the target construct and psychometric properties. Commonly used measures include the Professional Quality of Life Scale, compassion‐fatigue‐specific scales, and validated burnout measures when burnout is the target outcome.	[10, 13, 14, 21, 22]	Mainly Tier 1 evidence: systematic reviews/meta‐analyses supporting commonly used measurement tools; local validation should still be considered	Level 1b	A
	Targeted monitoring and early‐warning processes should be considered in high‐risk settings. Assessment should integrate symptom measures with contextual information such as traumatic exposure, workload, and access to recovery and support.	[12, 14, 15, 21, 22]	Tier 1 and Tier 2 evidence: guideline and systematic review evidence; downgraded due to limited direct evidence on early‐warning implementation	Level 1b	B

Proactive prevention strategies	Pre‐service and in‐service education may include recognition of compassion fatigue, empathic boundary setting, emotional regulation, mindfulness‐based stress management, self‐care, and pathways for psychological support.	[14, 20–24]	Mainly Tier 1 and Tier 2 evidence: systematic reviews/meta‐analyses; downgraded due to heterogeneity in intervention components and duration	Level 1b	B
	Team‐based preventive supports, including peer support, structured debriefing or reflection, supervision, and resilience‐oriented activities, may be considered for nurses repeatedly exposed to suffering, trauma, or death‐related events.	[10, 13, 20, 21, 24, 25]	Tier 1 and Tier 2 evidence: systematic reviews with variable confidence; downgraded because intervention models and implementation intensity differ	Level 1b	B

Multidimensional intervention strategies	Team‐level interventions such as peer support, supervision, structured reflection, resilience training, and group‐based stress‐management activities may be used when they are feasible and acceptable in the local setting.	[12–14, 23, 25]	Mainly Tier 1 and Tier 2 evidence: systematic reviews/meta‐analyses; downgraded due to heterogeneity and limited long‐term evidence	Level 1b	B
	Individual‐level interventions, including mindfulness‐based approaches, breathing and relaxation exercises, yoga, music‐based interventions, and other mind–body strategies, may be considered as supportive options. They should not replace professional psychological or medical care when clinically indicated.	[14, 20, 21, 23, 25]	Tier 1 and Tier 2 evidence: systematic reviews/meta‐analyses; downgraded due to heterogeneity in intervention format, outcome measures, and follow‐up duration	Level 1b	B
	Organizational strategies should address modifiable work conditions, including workload, scheduling, rest and recovery opportunities, access to psychological support, supportive leadership, and staff well‐being policies.	[12, 15, 18, 25]	Tier 2 and Tier 3 evidence: guideline and review evidence with indirect organizational implications; downgraded because direct effectiveness evidence is limited	Level 1b	B

Outcome evaluation and indicator framework	Core outcomes should include compassion fatigue or secondary traumatic stress, burnout, compassion satisfaction, and relevant well‐being and workforce outcomes such as stress, sleep, resilience, job satisfaction, and turnover intention.	[10, 13, 14, 22]	Mainly Tier 1 evidence: systematic reviews/meta‐analyses consistently reporting these outcomes	Level 1b	A
	Evaluation should include baseline and postintervention assessment and, where feasible, follow‐up assessment. High‐risk settings may require more frequent monitoring based on local protocols.	[12–15, 21]	Tier 1 and Tier 2 evidence: reviews and guideline evidence; downgraded because optimal timing and frequency remain uncertain	Level 1b	B
	Measurement tools should be selected according to the target construct, cultural context, psychometric properties, and feasibility. Cross‐cultural use should be supported by appropriate language adaptation and validity evidence.	[10, 13, 14, 21]	Mainly Tier 1 evidence: systematic reviews/meta‐analyses; recommendation strengthened because measurement standardization directly supports evaluation quality	Level 1b	A

Organizational support and implementation framework	Healthcare organizations should develop a defined compassion‐fatigue prevention and management pathway and integrate it into occupational health, nursing quality improvement, and staff well‐being systems.	[13, 15, 18, 20, 25]	Tier 1, Tier 2, and Tier 3 evidence: reviews, guidelines, and authoritative guidance; downgraded because direct implementation evidence is limited	Level 1b	B
	Healthcare organizations should provide accessible psychological support, appropriate supervision resources, reasonable scheduling and recovery opportunities, and culturally appropriate support for high‐risk nurses.	[10, 13, 18, 21, 25]	Tier 2 and Tier 3 evidence: systematic reviews supplemented by authoritative guidance; downgraded because direct implementation evidence is limited	Level 1b	B
	A supportive professional culture should reduce stigma, encourage help‐seeking and peer support, and normalize the use of available staff‐support resources through coordinated online and offline approaches where appropriate.	[10, 13, 14, 16, 22, 23]	Tier 1, Tier 2, and Tier 3 evidence: systematic review evidence supplemented by consensus/guidance; downgraded because culture‐change evidence is indirect	Level 1b	B

*Note:* Evidence statements were weighted hierarchically. Tier 1 included high‐quality clinical practice guidelines and systematic reviews or meta‐analyses with high or moderate AMSTAR 2 confidence; Tier 2 included moderate‐quality guidelines, systematic reviews with low AMSTAR 2 confidence, and directly relevant intervention studies; Tier 3 included expert consensus statements and evidence summaries from authoritative organizations; and Tier 4 included educational toolkits and other eligible gray literature. Lower tier sources were used mainly as supplementary evidence and were downgraded where appropriate. Recommendation grades also considered methodological quality, directness, consistency, feasibility, applicability, and clinical relevance. AMSTAR 2 = A Measurement Tool to Assess Systematic Reviews 2.

Abbreviations: RNAO, Registered Nurses’ Association of Ontario; SAMHSA, Substance Abuse and Mental Health Services Administration; STSC, Secondary Traumatic Stress Consortium.

## 4. Discussion

This evidence summary integrated 15 documents into 16 practice‐oriented statements across six domains. CF should be managed as a multilevel occupational issue rather than solely as an individual coping problem. Evidence was strongest and most direct for individual interventions and outcome measurement, whereas organizational implementation evidence was more indirect and heterogeneous.

### 4.1. Conceptual Clarification and Standardized Management of CF

Conceptual clarity is essential because CF, burnout, and STS share symptoms but reflect partly different occupational and trauma‐related processes [1, 26, 27]. Nursing services should define the construct being monitored and select measures and interventions accordingly.

The evidence supports standardized, context‐appropriate measurement, but no instrument is universally definitive. The Professional Quality of Life scale assesses compassion satisfaction, burnout, and STS, while other measures target related constructs. Instrument selection should consider the target construct, psychometric properties, language/cultural adaptation, and feasibility; repeated scores should be interpreted with clinical context rather than as stand‐alone diagnoses.

### 4.2. Risk Assessment and Early Identification in High‐Risk Nurses

Risk is greater among nurses repeatedly exposed to trauma, death, severe illness, high workload, or emotionally demanding care, including emergency, intensive‐care, oncology, psychiatric, and trauma‐related settings [10, 13, 20]. Assessment should therefore consider both occupational exposure and protective resources such as resilience, social support, and recovery opportunities.

Standardized assessment and targeted monitoring are supported in high‐risk settings, but evidence does not establish a universal screening interval, cutoff, or early‐warning algorithm. Nurse managers should combine validated measures with contextual indicators such as recent traumatic exposure, workload changes, absenteeism, turnover, and help‐seeking, with clear follow‐up pathways for elevated risk.

### 4.3. Multilevel Interventions: Evidence, Uncertainty, and Complementarity

The evidence supports complementary individual, team, and organizational strategies. Individual interventions are most frequently studied, whereas team‐ and organization‐level recommendations often derive from heterogeneous reviews, guidelines, or multicomponent programs. Implementation should therefore match support intensity to local risk, resources, and service context rather than shift responsibility solely to individual nurses.

#### 4.3.1. Individual‐Level Interventions

Individual strategies include mindfulness, relaxation and breathing exercises, yoga and other mind–body approaches, music‐based approaches, and structured self‐care. Reviews report improvements in some burnout, stress, or professional‐quality‐of‐life outcomes, but effects vary with intervention content, duration, comparators, and measurement [12, 14, 23, 25]. They should therefore supplement, not replace, responses to organizational determinants.

Self‐care education may support stress recognition, recovery, and earlier help‐seeking but should not imply individual responsibility for unsafe or chronically understaffed environments. Persistent, severe, or functionally impairing symptoms warrant professional psychological or medical support.

#### 4.3.2. Team‐Level Interventions

Team strategies include peer support, structured debriefing or reflection, supervision, resilience‐building, and group‐based stress‐management approaches. They may normalize help‐seeking and reduce isolation, but formats, facilitator roles, frequency, and outcomes vary substantially [13, 14, 25].

Because evidence is heterogeneous, programs should be selected according to exposure patterns, staffing, workforce preferences, and facilitation resources, with predefined outcomes and follow‐up so ineffective or burdensome components can be modified.

#### 4.3.3. Organizational‐Level Interventions

Organizational recommendations address workload, scheduling, recovery opportunities, psychological support, leadership, and staff well‐being policies. Guidelines and reviews support embedding CF prevention within occupational health and nursing governance rather than treating it as an optional wellness activity [12, 15, 18].

Nurse managers should define responsibility for screening and referral, ensure confidential support access, review workload and recovery opportunities in high‐risk units, and integrate staff well‐being indicators into routine management, particularly during repeated traumatic exposure or acute service pressure.

Direct causal evidence for specific organizational interventions remains limited because many recommendations derive from guidelines, observational evidence, or multicomponent programs. Organizational strategies should therefore be implemented with local evaluation rather than presented as universally proven interventions.

#### 4.3.4. Core Evidence Gaps and Optimization Priorities

Three gaps are prominent: short follow‐up limits conclusions about sustained effects; heterogeneous interventions and outcomes reduce comparability; and organization‐level strategies and implementation outcomes are less rigorously evaluated than individual approaches. These gaps help explain why most recommendations were graded B rather than A.

Future studies should specify intervention components, use construct‐appropriate standardized outcomes, extend follow‐up, and report implementation outcomes such as reach, adherence, acceptability, resources, and sustainability. In practice, high‐risk units should be prioritized and outcomes evaluated before wider scale‐up.

### 4.4. Evidence‐to‐Practice Translation

Translation requires assessment of workforce risk, support resources, staffing constraints, referral pathways, leadership responsibilities, and staff preferences. Because organizational culture and help‐seeking shape implementation, frontline nurses and managers should adapt interventions to local workflows. A staged process of local assessment, small‐scale implementation, evaluation, refinement, and broader rollout can identify context‐specific barriers before wider adoption.

### 4.5. Limitations

Several limitations should inform interpretation. First, English‐ and Chinese‐language eligibility may have introduced language bias. Second, despite searching multiple databases, evidence resources, organizational websites, and reference lists, relevant indexed or unpublished evidence may have been missed. Third, included systematic reviews may share primary studies; overlap was not formally quantified, so some evidence may have been represented more than once. Fourth, directness varied because some recommendations drew on broader nursing populations, specific specialties, or related outcomes such as burnout and STS rather than CF alone. Fifth, an educational toolkit and authoritative gray literature were used only as supplementary evidence, but their peer‐review status and methodological rigor differed from formal research publications. Sixth, although two reviewers used predefined integration rules and an explicit hierarchy, synthesis of heterogeneous evidence necessarily involved judgment and cannot eliminate subjectivity. Seventh, evidence arose from different healthcare systems and cultures, so recommendations concerning help‐seeking, psychological services, workload, staffing, leadership, and professional culture require local adaptation. Eighth, no formal certainty framework such as the Grading of Recommendations Assessment, Development and Evaluation (GRADE) approach was applied across the heterogeneous sources; JBI evidence levels and recommendation grades should not be interpreted as equivalent certainty ratings. Finally, patients, frontline nurses, managers, and other stakeholders did not directly prioritize or interpret the recommendations; future implementation should incorporate stakeholder consultation and local feasibility assessment.

## 5. Conclusion

This evidence summary synthesized 16 practice‐oriented statements from 15 documents. The findings support a multilevel approach combining conceptual clarification, risk assessment, individual and team interventions, outcome monitoring, and organizational support. Prevention should not rely solely on individual coping; nurse managers and healthcare organizations should also address workload, recovery opportunities, psychological‐support access, and a culture that supports help‐seeking.

The evidence provides a structured starting point rather than a universal protocol. Strategy selection should reflect clinical risk, workforce characteristics, resources, and organizational context, with local adaptation, stakeholder involvement, and prospective evaluation.

Future research should strengthen organizational‐intervention evidence, standardize outcomes, extend follow‐up, assess implementation and costs, and examine transferability across cultures and healthcare systems.

## 6. Relevance to Clinical Practice

For nurse managers, five linked actions emerge: identify high‐risk units and staff; use construct‐appropriate assessment; provide accessible individual and team support; address modifiable conditions such as workload, scheduling, recovery, and referral access; and monitor outcomes. A coordinated management pathway is preferable to isolated wellness activities, and local piloting and evaluation should precede broad adoption.

## Funding

No funding was received for this manuscript.

## Conflicts of Interest

The authors declare no conflicts of interest.

## Supporting Information

Additional supporting information can be found online in the Supporting Information section.

## Supporting information


**Supporting Information** Supporting File 1. Detailed Search Strategies. This file provides database‐specific search strategies, search fields, Boolean operators, search coverage, retrieval counts, organizational and gray‐literature search methods, reference‐list checking, and literature screening counts.

## Data Availability

The data that support the findings of this study are available from the corresponding author upon reasonable request.

## References

[1] Rauvola R. S. , Vega D. M. , and Lavigne K. N. , Compassion Fatigue, Secondary Traumatic Stress, and Vicarious Traumatization: A Qualitative Review and Research Agenda, Occupational Health Science. (2019) 3, no. 3, 297–336, 10.1007/s41542-019-00045-1.

[2] Coetzee S. K. and Klopper H. C. , Compassion Fatigue Within Nursing Practice: A Concept Analysis, Nursing and Health Sciences. (2010) 12, no. 2, 235–243, 10.1111/j.1442-2018.2010.00526.x. 20602697

[3] Schuster M. and Dwyer P. A. , Post-Traumatic Stress Disorder in Nurses: An Integrative Review, Journal of Clinical Nursing. (2020) 29, no. 15–16, 2769–2787, 10.1111/jocn.15288. 32372541

[4] Xie W. , Chen L. , Feng F. et al., The Prevalence of Compassion Satisfaction and Compassion Fatigue Among Nurses: A Systematic Review and Meta-Analysis, International Journal of Nursing Studies. (2021) 120, 10.1016/j.ijnurstu.2021.103973. 34102372

[5] Yi L. , Hu S. , Liao M. et al., Prevalence and Related Factors of Compassion Fatigue Among Registered Nurses and Nursing Students During the Internship: A Systematic Review and Meta-Analysis, BMC Nursing. (2024) 23, no. 1, 10.1186/s12912-024-02597-2. PMC11687103 39736724

[6] Sheppard K. , Compassion Fatigue Among Registered Nurses: Connecting Theory and Research, Applied Nursing Research. (2015) 28, no. 1, 57–59, 10.1016/j.apnr.2014.10.007. 25434861

[7] Rodney T. , Heidari O. , Miller H. N. , Thornton C. P. , Jenkins E. , and Kang H. K. , Posttraumatic Stress Disorder in Nurses in the United States: Prevalence and Effect on Role, Journal of Nursing Management. (2022) 30, no. 1, 226–233, 10.1111/jonm.13478. 34590765

[8] Wolf L. A. , Delao A. M. , Perhats C. , Clark P. R. , Edwards C. , and Frankenberger W. D. , Traumatic Stress in Emergency Nurses: Does Your Work Environment Feel Like a War Zone?, International Emergency Nursing. (2020) 52, 10.1016/j.ienj.2020.100895. 32795958

[9] Amberson T. and Quarry C. , Understanding Traumatic Stress in Emergency Nurses: A Systematic Review, Journal of Advanced Nursing. (2025) 81, no. 10, 6261–6280, 10.1111/jan.16809. 39991964

[10] Ortega-Campos E. , Vargas-Román K. , Velando-Soriano A. et al., Compassion Fatigue, Compassion Satisfaction, and Burnout in Oncology Nurses: A Systematic Review and Meta-Analysis, Sustainability. (2020) 12, no. 1, 10.3390/su12010072.

[11] Xu Z. , Zhao B. , Zhang Z. et al., Prevalence and Associated Factors of Secondary Traumatic Stress in Emergency Nurses: A Systematic Review and Meta-Analysis, European Journal of Psychotraumatology. (2024) 15, no. 1, 10.1080/20008066.2024.2321761. PMC10911249 38426665

[12] Hsu H.-C. , Lee H.-F. , Hung H.-M. et al., Effectiveness of Individual-Based Strategies to Reduce Nurse Burnout: An Umbrella Review, Journal of Nursing Management. (2024) 2024, 8544725–13, 10.1155/2024/8544725. PMC11918503 40224800

[13] Patole S. , Pawale D. , and Rath C. , Interventions for Compassion Fatigue in Healthcare Providers: A Systematic Review of Randomised Controlled Trials, Healthcare. (2024) 12, no. 2, 10.3390/healthcare12020171. PMC10815881 38255060

[14] Zhang H. , Xia Z. , Yu S. , Shi H. , Meng Y. , and Dator W. L. , Interventions for Compassion Fatigue, Burnout, and Secondary Traumatic Stress in Nurses: A Systematic Review and Network Meta-Analysis, Nursing and Health Sciences. (2025) 27, no. 1, 10.1111/nhs.70042. 39887608

[15] Registered Nurses’ Association of Ontario , Preventing and Mitigating Nurse Fatigue in Health Care: Healthy Work Environments Best Practice Guideline, 2011, https://rnao.ca/bpg/guidelines/preventing-and-mitigating-nurse-fatigue-health-care.

[16] Secondary Traumatic Stress Consortium , Best Practices for Addressing Secondary Traumatic Stress: Principles for Individuals & Organizations, 2021, https://www.stsconsortium.com/consensus-main-page.

[17] Bride B. E. , Sprang G. , Hendricks A. et al., Principles for Secondary Traumatic Stress-Responsive Practice: An Expert Consensus Approach, Psychological Trauma. Theory, Research, Practice, and Policy. (2024) 16, no. 8, 1301–1308, 10.1037/tra0001575. 37650802

[18] Substance Abuse and Mental Health Services Administration , Tips for Healthcare Professionals: Coping With Stress and Compassion Fatigue, 2020, https://library.samhsa.gov/sites/default/files/PEP20-01-01-016_508.pdf.

[19] Enakhena C. , Educational Tool Kit for Mental Health Nurses to Combat Compassion Fatigue (Doctor of Nursing Practice Project), 2022, Walden University ScholarWorks, https://scholarworks.waldenu.edu/dissertations/13336/.

[20] Şimşek B. , Özer Z. , and Buldukoğlu K. , Investigation of the Effect of Secondary Traumatic Stress on Psychological Resilience in Emergency Nurses: A Systematic Review, Journal of Clinical Nursing. (2025) 34, no. 7, 2564–2572, 10.1111/jocn.17688. PMC12181162 40013486

[21] Yu J. , Chang Y. , Li Y. , Guo H. , and Cao H. , Meta Analysis of Mindfulness Therapy in Improving Compassion Fatigue of Nurses, Occupation and Health. (2023) 39, no. 15, 2123–2128.

[22] Lee M. and Cha C. , Interventions to Reduce Burnout Among Clinical Nurses: Systematic Review and Meta-Analysis, Scientific Reports. (2023) 13, no. 1, 10.1038/s41598-023-38169-8. PMC10325963 37414811

[23] Wang Q. , Wang F. , Zhang S. , Liu C. , Feng Y. , and Chen J. , Effects of a Mindfulness-Based Interventions on Stress, Burnout in Nurses: A Systematic Review and Meta-Analysis, Frontiers in Psychiatry. (2023) 14, 10.3389/fpsyt.2023.1218340. PMC10434780 37599884

[24] Chen X. , Chen M. , Zheng H. et al., Effects of Psychological Intervention on Empathy Fatigue in Nurses: A Meta-Analysis, Frontiers in Public Health. (2022) 10, 10.3389/fpubh.2022.952932. PMC9614432 36311568

[25] Jung S.-E. , Ha D.-J. , Park J.-H. et al., The Effectiveness and Safety of Mind-Body Modalities for Mental Health of Nurses in Hospital Setting: A Systematic Review, International Journal of Environmental Research and Public Health. (2021) 18, no. 16, 10.3390/ijerph18168855. PMC8393251 34444604

[26] Figley C. R. , Compassion Fatigue: Psychotherapists’ Chronic Lack of Self Care, Journal of Clinical Psychology. (2002) 58, no. 11, 1433–1441, 10.1002/jclp.10090. 12412153

[27] Nolte A. G. W. , Downing C. , Temane A. , and Hastings-Tolsma M. , Compassion Fatigue in Nurses: A Metasynthesis, Journal of Clinical Nursing. (2017) 26, no. 23–24, 4364–4378, 10.1111/jocn.13766. 28231623

