# Supplementary material for: Prevention and Management of Compassion Fatigue in Clinical Nurses: An Evidence Summary
Source: J Nurs Manag. 2026 Nov 2;2026:5171257. doi: 10.1155/jonm/5171257 (PMC13728450; doi:10.1155/jonm/5171257)
Supplement: Supplementary file 1 [file JONM-2026-5171257-s001.docx]

**Supplementary File 1: Detailed Search Strategies for the Prevention and Management of Compassion Fatigue in Clinical Nurses**

English-language version prepared for journal submission.

# Search documentation and scope

Source documentation: The retained search records establish a common search coverage end date of 30 June 2025 and the database-specific expressions reproduced or translated below. For organization websites and repositories, exact historical search strings were not always preserved; no undocumented query has been reconstructed.

Search coverage: From database/resource inception through 30 June 2025.

Search end date: 30 June 2025. This is the documented coverage end date, not verification of a separate execution date for each source.

Language eligibility: English and Chinese. Language eligibility was applied during study selection rather than uniformly as a database filter.

Evidence types targeted: Guidelines, expert consensus statements, systematic reviews, meta-analyses, evidence summaries, eligible intervention studies, and authoritative grey literature.

Record management: Retrieved records were imported into EndNote; 117 duplicates were removed.

Search yield: Bibliographic databases/evidence resources yielded 1,476 records (Chinese-language databases, n = 47; English-language databases/resources, n = 1,429). Targeted organizational/repository sources added five records, giving 1,481 before deduplication. Backward reference checking yielded five further reports for full-text assessment outside the 1,481.

# S1. Search sources, fields, limits and yields

| **Source** | **Platform / field** | **Coverage end** | **Records** | **Notes / scope** |
| --- | --- | --- | --- | --- |
| CNKI | Subject | 30 Jun 2025 | 17 | Inception to 30 Jun 2025 |
| VIP Chinese Scientific and Technological Journal Database | Title or keyword | 30 Jun 2025 | 7 | Inception to 30 Jun 2025 |
| Chinese Biomedical Literature Database (CBM) | All fields; unweighted/expanded | 30 Jun 2025 | 13 | Inception to 30 Jun 2025 |
| Wanfang Data | Title or keyword | 30 Jun 2025 | 10 | Inception to 30 Jun 2025 |
| PubMed | MeSH + Title/Abstract | 30 Jun 2025 | 28 | Inception to 30 Jun 2025 |
| Embase | Title/Abstract | 30 Jun 2025 | 225 | Inception to 30 Jun 2025 |
| Cochrane Library | Title/Abstract/Keyword + MeSH | 30 Jun 2025 | 233 | Inception to 30 Jun 2025 |
| Web of Science Core Collection | Topic (TS) | 30 Jun 2025 | 578 | Inception to 30 Jun 2025 |
| ClinicalKey | Comprehensive search | 30 Jun 2025 | 2 | Inception to 30 Jun 2025 |
| Ovid + JBI resources | Title/Abstract/Keyword | 30 Jun 2025 | 64 | Journals@Ovid Full Text and JBI Best Practice |
| Ovid multi-file psychology/medical search | Title/Abstract/Keyword | 30 Jun 2025 | 229 | APA PsycArticles, Books@Ovid, Ovid MEDLINE, APA PsycINFO |
| APA PsycINFO (independent search) | Title/Abstract | 30 Jun 2025 | 70 | Psychology-focused search |
| Targeted organizational/grey-literature sources | Organization-site/internal search | 30 Jun 2025 | 5 | RNAO, STSC, APA, SAMHSA, Walden ScholarWorks |

Note. Each search covered database/resource inception through the stated end date unless otherwise shown. The language eligibility criterion was applied at screening; it was not uniformly applied as a database filter.

# S2. Chinese-language database strategies (English translations)

The four strategies below are English translations of Chinese-language searches documented for CNKI, VIP, CBM and Wanfang Data. They describe the original concepts, logic and field specifications for editorial review; they are not verbatim executable commands for Chinese interfaces. Counts correspond to the documented searches executed in Chinese. The original Chinese strings remain preserved in the search documentation and have not been replaced by newly executed English queries.

## S2.1 China National Knowledge Infrastructure (CNKI)

Field: Subject. Records retrieved: 17.

(compassion fatigue OR empathy fatigue OR compassion weariness OR compassion fatigue (variant) OR secondary trauma OR secondary traumatization OR secondary traumatic stress OR emotional exhaustion OR emotional fatigue) AND (clinical nurse OR nurse OR primary nurse OR new nurse OR ward nurse OR ICU nurse OR nursing personnel) AND (systematic review OR systematic review and meta-analysis OR systematic review with meta-analysis OR systematic review study OR overview of systematic reviews OR meta-analysis OR guideline OR guideline interpretation OR guideline research OR diagnosis and treatment guideline OR treatment guideline OR relevant guideline OR randomized controlled trial OR randomized controlled trial study OR randomized controlled trial method OR random OR randomized control OR RCT OR evidence summary OR consensus)

## S2.2 VIP Chinese Scientific and Technological Journal Database

Field: Title or keyword. Records retrieved: 7.

((((((((Title/Keyword=empathy fatigue OR Title/Keyword=compassion weariness) OR Title/Keyword=compassion fatigue) OR Title/Keyword=compassion fatigue (variant)) OR Title/Keyword=emotional exhaustion) OR Title/Keyword=emotional fatigue) OR (Title/Keyword=secondary traumatization OR Title/Keyword=secondary traumatic stress)) AND (Title/Keyword=nurse OR Title/Keyword=clinical nursing practitioner)) AND (((((((((((Title/Keyword=systematic review OR Title/Keyword=evaluation of system) OR Title/Keyword=system assessment) OR Title/Keyword=system evaluation) OR Title/Keyword=systematic assessment) OR Title/Keyword=systematic evaluation) OR Title/Keyword=systematic review) OR Title/Keyword=systematical review) OR Title/Keyword=systematic review) OR Title/Keyword=meta-analysis) OR Title/Keyword=guideline) OR Title/Keyword=randomized controlled trial))

## S2.3 Chinese Biomedical Literature Database (CBM)

Field: All fields; unweighted/expanded. Records retrieved: 13.

("nurse"[unweighted:expanded]) AND (("compassion fatigue"[unweighted:expanded]) OR emotional fatigue OR emotional exhaustion OR compassion weariness) AND (consensus OR evidence summary OR randomized controlled trial OR Randomized Controlled Trial OR guideline OR Guidebook OR meta-analysis)

## S2.4 Wanfang Data Knowledge Service Platform

Field: Title or keyword. Records retrieved: 10.

(compassion fatigue OR empathy fatigue OR compassion weariness OR compassion fatigue (variant) OR secondary trauma OR secondary traumatization OR secondary traumatic stress OR emotional exhaustion OR emotional fatigue) AND (clinical nurse OR nurse OR nursing personnel) AND (systematic review OR meta-analysis OR overview of systematic reviews OR guideline OR randomized controlled trial OR evidence summary)

# S3. English-language database/resource strategies

## S3.1 PubMed

Field: MeSH terms and title/abstract. Records retrieved: 28.

((("Compassion Fatigue"[Mesh]) OR (Fatigue, Compassion[Title/Abstract] OR Secondary Trauma[Title/Abstract] OR Trauma, Secondary[Title/Abstract] OR Secondary Traumatization[Title/Abstract] OR Traumatization, Secondary[Title/Abstract] OR Vicarious Trauma[Title/Abstract] OR Trauma, Vicarious[Title/Abstract] OR Secondary Traumatic Stress[Title/Abstract] OR Traumatic Stress, Secondary[Title/Abstract] OR Vicarious Traumatization[Title/Abstract])) OR (("Emotional Exhaustion"[Mesh]) OR (Emotional Exhaustions[Title/Abstract] OR Exhaustion, Emotional[Title/Abstract] OR Fatigue, Emotional[Title/Abstract]))) AND (("Nurses"[Mesh]) OR (Clinical Nurses[Title/Abstract] OR Nursing Staff[Title/Abstract])) AND (Systematic Review[Title/Abstract] OR Guideline[Title/Abstract] OR Randomized Controlled Trial[Title/Abstract] OR evidence summar*[Title/Abstract] OR Randomiz*[Title/Abstract])

## S3.2 Embase

Field: Title/abstract (ti,ab). Records retrieved: 225.

#1 fatigue, compassion:ab,ti OR secondary trauma:ab,ti OR secondary traumas:ab,ti OR trauma, secondary:ab,ti OR secondary traumatization:ab,ti OR secondary traumatizations:ab,ti OR traumatizations, secondary:ab,ti OR vicarious trauma:ab,ti OR traumas, vicarious:ab,ti OR secondary traumatic:ab,ti OR stresses, secondary traumatic:ab,ti OR traumatic stress, secondary:ab,ti OR traumatization, vicarious:ab,ti OR compassion fatigue:ab,ti OR emotional exhaustions:ab,ti OR exhaustion, emotional:ab,ti OR emotional fatigue:ab,ti OR fatigue, emotional:ab,ti OR emotional exhaustion:ab,ti
#2 nurses:ab,ti OR clinical nurses:ab,ti OR nursing staff:ab,ti
#3 systematic review:ab,ti OR guideline:ab,ti OR randomized controlled trial:ab,ti OR meta-analysis:ab,ti OR evidence summar*:ab,ti OR consensus:ab,ti OR randomiz*:ab,ti
#4 #1 AND #2 AND #3

## S3.3 Cochrane Library

Field: Title/abstract/keyword (ti,ab,kw) and MeSH. Records retrieved: 233.

#1 MeSH descriptor: [Compassion Fatigue] explode all trees
#2 Trauma, Secondary:ti,ab,kw OR Secondary Trauma:ti,ab,kw OR Secondary Traumatization:ti,ab,kw OR Traumatization, Secondary:ti,ab,kw OR Vicarious Trauma:ti,ab,kw OR Trauma, Vicarious:ti,ab,kw OR Secondary Traumatic Stress:ti,ab,kw OR Traumatic Stress, Secondary:ti,ab,kw OR Vicarious Traumatization:ti,ab,kw
#3 Nurses:ti,ab,kw OR Clinical Nurses:ti,ab,kw OR Nursing Staff:ti,ab,kw OR clinical nurs*:ti,ab,kw
#4 Systematic Review:ti,ab,kw OR Guideline:ti,ab,kw OR Randomized Controlled Trial:ti,ab,kw OR evidence summar*:ti,ab,kw OR Randomiz*:ti,ab,kw
#5 #1 OR #2
#6 #3 AND #4 AND #5

## S3.4 Web of Science Core Collection

Field: Topic (TS). Records retrieved: 578.

TS=(Trauma, Secondary OR Secondary Traumatization OR Secondary Traumatizations OR Traumatizations, Secondary OR Vicarious Trauma OR Traumas, Vicarious OR Secondary Traumatic OR Stresses, Secondary Traumatic OR Traumatic Stress, Secondary OR Vicarious Traumatization OR Compassion Fatigue OR Emotional Exhaustions OR Exhaustion, Emotional OR Emotional Fatigue OR Fatigue, Emotional OR emotional exhaustion) AND TS=(Nurses OR Clinical Nurses OR Nursing Staff) AND TS=(Systematic Review OR Guideline OR Randomized Controlled Trial OR meta-analysis OR evidence summar* OR consensus OR Randomiz*)

## S3.5 ClinicalKey

Field: Comprehensive search. Records retrieved: 2.

compassion fatigue nurses intervention

## S3.6 Ovid + Joanna Briggs Institute resources

Field: Title/abstract/keyword (ti,ab,kw). Records retrieved: 64. Resources: Journals@Ovid Full Text; JBI Best Practice.

((Emotional Exhaustion OR Emotional Exhaustions OR Exhaustion, Emotional OR Fatigue, Emotional).ti,ab,kw. OR (Compassion Fatigue OR Fatigue, Compassion OR Secondary Trauma OR Trauma, Secondary OR Secondary Traumatization OR Traumatization, Secondary OR Vicarious Trauma).ti,ab,kw.) AND (Systematic Review OR Guideline OR Randomized Controlled Trial OR meta-analysis OR evidence summar*).ti,ab,kw. AND (Nurses OR Clinical Nurses OR Nursing Staff).ti,ab,kw.

## S3.7 Ovid multi-file psychology/medical search

Field: Title/abstract/keyword (ti,ab,kw). Records retrieved: 229. Resources: APA PsycArticles; Books@Ovid; Ovid MEDLINE; APA PsycINFO.

((Emotional Exhaustion OR Emotional Exhaustions OR Exhaustion, Emotional OR Fatigue, Emotional).ti,ab,kw. OR (Compassion Fatigue OR Fatigue, Compassion OR Secondary Trauma OR Trauma, Secondary OR Secondary Traumatization OR Traumatization, Secondary OR Vicarious Trauma).ti,ab,kw.) AND (Systematic Review OR Guideline OR Randomized Controlled Trial OR meta-analysis OR evidence summar*).ti,ab,kw. AND (Nurses OR Clinical Nurses OR Nursing Staff).ti,ab,kw.

## S3.8 APA PsycINFO (independent search)

Field: Title/abstract (ti,ab). Records retrieved: 70.

((Compassion Fatigue OR Fatigue, Compassion OR Secondary Trauma OR Trauma, Secondary OR Secondary Traumatization OR Traumatization, Secondary OR Vicarious Trauma).ti,ab. OR (Emotional Exhaustion OR Emotional Exhaustions OR Exhaustion, Emotional OR Fatigue, Emotional).ti,ab.) AND (Systematic Review OR Guideline OR Randomized Controlled Trial OR meta-analysis OR evidence summar*).ti,ab. AND (Nurses OR Clinical Nurses OR Nursing Staff).ti,ab.

# S4. Targeted organizational and grey-literature searches

The following organizational sites and repository were searched or navigated for directly relevant guidance, consensus, or educational material that may be inconsistently indexed in bibliographic databases. The archived records identify the source, topic focus, item and yield. Because exact historical site-query syntax was not verifiable for these five sources, reconstructed Boolean searches are not presented.

| Source | Documented focus / approach | Identified record | n |
| --- | --- | --- | --- |
| Registered Nurses' Association of Ontario (RNAO) | Targeted site search/navigation: nurse fatigue and compassion-fatigue guidance. | Preventing and Mitigating Nurse Fatigue in Health Care | 1 |
| Secondary Traumatic Stress Consortium (STSC) | Targeted site search/navigation: secondary-traumatic-stress practice guidance. | Best Practices for Addressing Secondary Traumatic Stress: Principles for Individuals & Organizations | 1 |
| American Psychological Association (APA) | Targeted organization/publisher search: secondary-traumatic-stress-responsive practice. | Principles for Secondary Traumatic Stress-Responsive Practice: An Expert Consensus Approach | 1 |
| Substance Abuse and Mental Health Services Administration (SAMHSA) | Targeted site search: healthcare-professional stress and compassion fatigue guidance. | Tips for Healthcare Professionals: Coping with Stress and Compassion Fatigue | 1 |
| Walden University ScholarWorks | Targeted repository search: educational toolkit for mental health nurses. | Educational Tool Kit for Mental Health Nurses to Combat Compassion Fatigue | 1 |

# S5. Backward reference-list checking

Backward reference-list checking was performed for potentially eligible and included sources. Five additional reports were identified through this process and were taken directly to full-text eligibility assessment. These five reports were additional to the 1,481 records identified through bibliographic and targeted organizational searches.

# S6. Screening counts linked to Figure 1

| Screening stage | Number |
| --- | --- |
| Records before deduplication | 1,481 |
| Duplicates removed | 117 |
| Title/abstract records screened | 1,364 |
| Title/abstract exclusions | 956 |
| Potentially relevant reports after title/abstract screening | 408 |
| Additional reports from reference-list checking | 5 |
| Full-text reports assessed | 413 |
| Full-text exclusions | 398 |
| — Ineligible content | 333 |
| — Ineligible publication/research type | 55 |
| — Full text unavailable | 10 |
| Documents included in final synthesis | 15 |

Reconciliation: 1,476 + 5 = 1,481; 1,481 - 117 = 1,364; 1,364 - 956 = 408; 408 + 5 = 413; 413 - 398 = 15; and 333 + 55 + 10 = 398.
